# Primary hepatic angiosarcoma in an elderly patient

**DOI:** 10.4322/acr.2021.364

**Published:** 2022-03-02

**Authors:** Mayur Parkhi, Aravind Sekar

**Affiliations:** 1 Post Graduate Institute of Medical Education and Research, Department of Histopathology, Chandigarh, India

**Keywords:** Hepatomegaly, Hemangiosarcoma, Immunohistochemistry

Primary hepatic angiosarcoma is a high-grade mesenchymal tumor with very aggressive behavior, and accounts for 0.1-2% of all liver malignancies.^1^ Most of the time, patients present with abdominal pain, fatigue, weight loss, hepatosplenomegaly, ascites, jaundice, and anemia. This neoplasm is known for intraperitoneal rupture. Poor prognostic factors include older age, large tumor size, and a high Ki-67 index. It occurs in association with exposure to known chemical carcinogens such as vinyl chloride monomer, thorotrast, anabolic steroids, and arsenic. However, in 75% of cases, the etiology is not known. The current molecular data revealed KRAS mutations in sporadic cases and TP53 mutations in vinyl chloride-related cases.^2^ Primary hepatic angiosarcoma may have different growth patterns on imaging. It includes 1) Multiple nodules, 2) Large dominant mass, 3) Mixed pattern of dominant mass with nodules, and 4) Diffuse infiltrating micronodular mass.^3^ The liver biopsy is the gold standard for confirming the diagnosis of primary hepatic angiosarcoma. Microscopically, the lesion is heterogeneous, ranging from well-defined anastomotic vessels (Vaso formative) to solid sheets of epithelioid or spindled cells; some tumors may show mixed patterns. Immunohistochemically, ERG (erythroblast transformation specific-related gene) and endothelial markers such as CD31 and CD34 show expression, indicating the endothelial origin of the tumor. D2-40 and CD10 may also show positivity. Of note, the epithelioid variant may show keratin expression. Surgical resection seems the best therapeutic option, leading to a median overall survival of between 17 and 19 months.^4^

**Figure 1:**
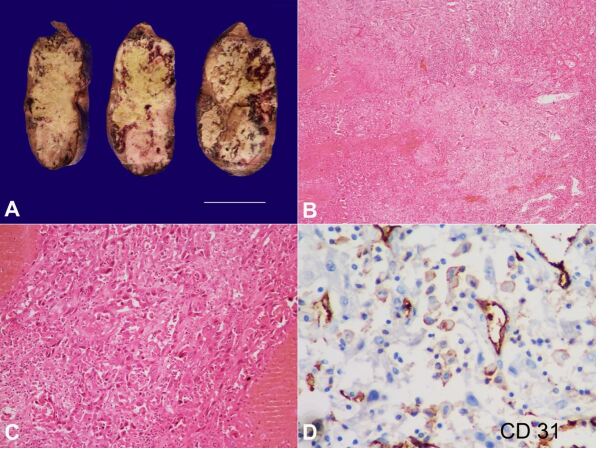
**A –** Gross view of the surgical specimen. Slices of right hepatectomy specimen showing the mixed growth pattern with dominant mass, measuring 18x10x5cm and nodules of variable size. Cut surface of the tumor showing pale white to yellowish firm areas with areas of hemorrhage and necrosis (scale bar = 15 cm); **B –** Microscopy showing an infiltrating tumor with areas of hemorrhage and fibrosis. (H&E,40 x); **C –** Higher magnification depicting the large, pleomorphic tumor cells lining the abortive sinusoids. (H&E, 200 x); **D –** Immunohistochemistry for CD31 showing membranous positivity in the tumor cells (400x).

We describe gross examination and supportive microscopic findings of primary hepatic angiosarcoma in a 66-year-old gentleman with no predisposing environmental risk factors. He presented with fatigue, weight loss, jaundice, abdominal pain, and swelling in the right hypochondrium. On investigation, he was found to have raised serum levels of ammonia (183.8 µmol/L; RR: 11-32 µmol/L), AST (89.5 U/L; RR: 5-40 U/L), ALT (77.6 U/L; RR: 7-56 U/L), ALP (410 IU/L; RR: 44-147 IU/L). Viral markers were negative, and the coagulation profile was within the normal limit. Hepatocellular carcinoma and Cholangiocarcinoma involving the right lobe were differential diagnoses based on clinical and imaging studies. Right hepatectomy was done and submitted for histopathological examination. Gross examination showed a dominant mass, measuring 18x10x5cm, and nodules of variable size. The liver capsule was smooth, shiny and there was no breach by the tumor. The cut surface of the tumor appeared variegated and showed pale white to yellowish firm areas with intervening areas of hemorrhage and necrosis (Figure 1 A). The adjacent native liver parenchyma was non-cirrhotic. Microscopically, the tumor was ill-defined, and the cells were arranged along with abortive vascular spaces and as clusters (Figure 1B). The tumor cells displayed moderate to marked degrees of nuclear pleomorphism with oval to plump to spindle-shaped vesicular nuclei, prominent nucleoli, and moderate eosinophilic cytoplasm (Figure 1C). Mitotic activity was brisk (>8 per 10 high power fields). Lymphovascular emboli were seen. Areas of necrosis and hemorrhage were present. The tumor cells exhibited membranous positivity for CD31 (Figure 1D) and CD34 immunomarkers. HepPar1, Arginase, and Keratin 7 were negative. Based on the light microscopy and immunohistochemistry findings, the diagnosis of primary hepatic angiosarcoma was offered.
